# New Irradiation Method with Indocyanine Green-Loaded Nanospheres for Inactivating Periodontal Pathogens

**DOI:** 10.3390/ijms18010154

**Published:** 2017-01-13

**Authors:** Yasuyuki Sasaki, Jun-ichiro Hayashi, Takeki Fujimura, Yuki Iwamura, Genta Yamamoto, Eisaku Nishida, Tasuku Ohno, Kosuke Okada, Hiromitsu Yamamoto, Takeshi Kikuchi, Akio Mitani, Mitsuo Fukuda

**Affiliations:** 1 Department of Periodontology, School of Dentistry, Aichi Gakuin University, Nagoya, Aichi 464-8651, Japan; ag143d09@dpc.agu.ac.jp (Y.S.); takeki@dpc.agu.ac.jp (T.F.); yukiwa@dpc.agu.ac.jp (Y.I.); genta@dpc.agu.ac.jp (G.Y.); enishida@dpc.agu.ac.jp (E.N.); ag143d03@dpc.agu.ac.jp (T.O.); ag133d03@dpc.agu.ac.jp (K.O.); tkikuchi@dpc.agu.ac.jp (T.K.); minita@dpc.agu.ac.jp (A.M.); fukuda-m@dpc.agu.ac.jp (M.F.); 2 Division of Periodontal Health Promotion, Dental Hospital, Aichi Gakuin University, Nagoya, Aichi 464-8651, Japan; 3 Department of Pharmaceutical Engineering, School of Pharmacology, Aichi Gakuin University, Nagoya, Aichi 464-8650, Japan; hiromitu@dpc.agu.ac.jp

**Keywords:** photodynamic therapy, indocyanine green, diode laser

## Abstract

Antimicrobial photodynamic therapy (aPDT) has been proposed as an adjunctive strategy for periodontitis treatments. However, use of aPDT for periodontal treatment is complicated by the difficulty in accessing morphologically complex lesions such as furcation involvement, which the irradiation beam (which is targeted parallel to the tooth axis into the periodontal pocket) cannot access directly. The aim of this study was to validate a modified aPDT method that photosensitizes indocyanine green-loaded nanospheres through the gingivae from outside the pocket using a diode laser. To establish this trans-gingival irradiation method, we built an in vitro aPDT model using a substitution for gingivae. Irradiation conditions and the cooling method were optimized before the bactericidal effects on *Porphyromonas gingivalis* were investigated. The permeable energy through the gingival model at irradiation conditions of 2 W output power in a 50% duty cycle was comparable with the transmitted energy of conventional irradiation. Intermittent irradiation with air cooling limited the temperature increase in the gingival model to 2.75 °C. The aPDT group showed significant bactericidal effects, with reductions in colony-forming units of 99.99% after 5 min of irradiation. This effect of aPDT against a periodontal pathogen demonstrates the validity of trans-gingival irradiation for periodontal treatment.

## 1. Introduction

Periodontitis is caused by anaerobic bacteria, which form a biofilm on tooth surfaces or in the periodontal pocket. These actions provoke an excessive and aggressive immune reaction in the host, and cause collateral damage to periodontal tissues [1]. Removal of the biofilm and elimination of periodontal pathogens from the periodontal pocket is the main purpose of treatment for this disease [2]. Scaling and root-planing (SRP) is a non-surgical method of mechanical debridement to eliminate calculus, plaque, and contaminated root cementum/dentine from the periodontal pocket and usually results in significant clinical improvement. However, complete eradication of pathogenic bacteria from areas that are inaccessible to periodontal instruments is difficult. Matia et al. reported that even after non-surgical mechanical debridement, residual calculus was present in 37.7% of furcation areas [3].

As an adjunct to SRP in inaccessible areas, various other treatment strategies have been evaluated [4,5,6,7], including the use of lasers. CO_2_, Nd:YAG, and Er:YAG lasers have been shown to be effective modalities in non-surgical periodontal therapy, with clinical effects including the removal of calculus, detoxification of the root surface, and bacterial lysis [8,9]. However, these high-level lasers can produce unacceptable thermal damage to the dental pulp and periodontal tissues [10].

Recently, antimicrobial photodynamic therapy (aPDT) has been proposed as an alternative strategy to conventional adjunctive treatment for SRP [11,12,13,14]. The general mechanism of aPDT is chemical sterilization by the highly reactive oxygen species (ROS) formed by irradiation of a photosensitizer with a low-level laser of appropriate wavelength. This ROS sterilization has been shown to be effective even against resistant bacterial species [15,16]. In a recent study, we validated the principle of using aPDT for periodontal therapy. We employed an 810-nm diode laser with indocyanine green-loaded nanospheres coated with chitosan (ICG-Nano/c) to demonstrate nano-spherization, positive charging of the chitosan coating of the photosensitizer, and resultant improved antibacterial effects of aPDT [17].

**Scheme 1:**
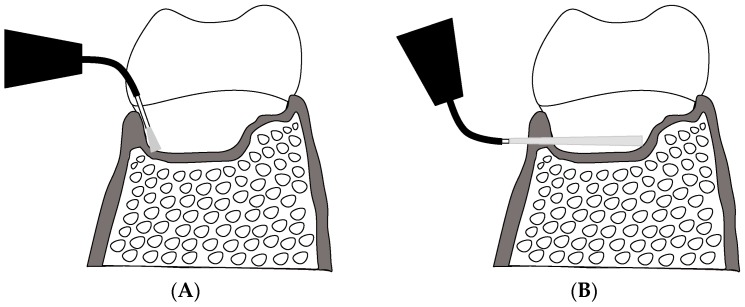
Advantages of a novel irradiation method for extending aPDT into inaccessible areas (schematic). (**A**) Conventional irradiation method via the periodontal pocket (intra-pocket irradiation). Sufficient light cannot be delivered to inaccessible areas (e.g., furcation) due to the irradiation direction being parallel to the tooth axis; (**B**) Novel trans-gingival irradiation from outside the periodontal pocket (external irradiation): light passing through the gingiva can penetrate deep into inaccessible areas.

Furcation involvement is a formidable challenge in the treatment of periodontitis, because of the morphological complexity and irregular structure of the bone defects in these areas [18]. Conventional irradiation methods for aPDT can produce insufficient sterilization effects in furcations (Scheme 1A). This is because the direction of insertion of the optical fiber into the periodontal pocket—From the gingival margin and parallel to the tooth axis—Limits the amount of light penetrating into the furcation, and thus the photosensitizer is excited only partially in these areas. A novel irradiation method, in which this directional limitation is overcome, may deliver sufficient light to the furcation to resolve this problem (Scheme 1B).

Most biological soft tissues have low light absorption properties in the near infrared (NIR) spectral regions, especially between 600–1300 nm [19]. This spectral range is called the “tissue optical window” [20], within which light can be transmitted into deep layers of soft tissue without significant absorption [21]. The penetration depth into soft tissue for wavelengths <650 nm is only 3.0–3.5 mm, whereas for NIR light it can be ≤6 mm [22]. The diode laser used in the present study emitted light at 810 nm, which can pass several millimeters into soft tissues.

In conjunction with these lasers, indocyanine green (ICG) is used commonly as a photosensitizer because of its wide band of optical absorption (from 600 to >800 nm) and its absorption peak at 805 nm in plasma protein [23]. Recently, ICG and its derivatives have also been applied as NIR fluorescent probes for optical imaging because of their optical properties. That is, their excitation and fluorescence wavelengths fall within the tissue optical window [24].

Thus, we hypothesized that trans-gingival irradiation using a diode laser could deliver sufficient energy to excite ICG in sub-gingival areas such as the furcation, where conventional methods for intra-pocket irradiation fail to penetrate. The purpose of the present study was to ascertain, in an in vitro model, if aPDT using external irradiation methods can produce efficacious bactericidal effects. We investigated the antibacterial effects of ICG-Nano/c activated by this new irradiation method, and describe the optimization of conditions for its future clinical application.

## 2. Results

### 2.1. Selection of Gingival Model

**Figure 1:**
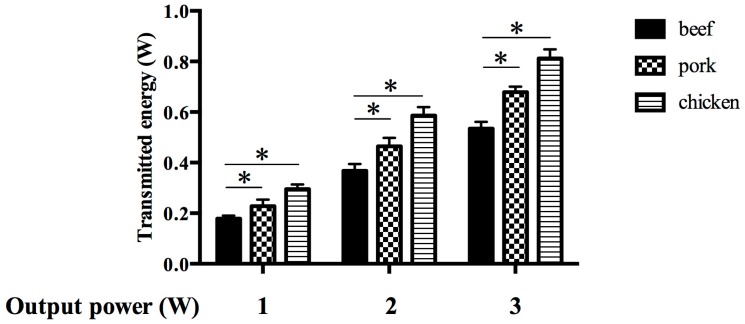
Selection of a gingival model according to the permeability of the laser. Bars denote the permeable energies transmitted through three types of raw meat with different contents of myoglobin. The tissue with the lowest permeability was selected as the best gingival model. Data are the mean ± standard deviation. * *p* < 0.05 (*n* = 3).

To select a suitable tissue material for a model mimicking human gingiva, three types of tissue slices were prepared and the permeable energies through these were measured during diode laser irradiation. The permeable energies through each type of tissue were increased proportional to peak power output (Figure 1). The energy through the fresh beef slice was the lowest in all settings of power output. Low permeability of energy was the most important property for the gingival model, so beef was selected as the most suitable material for use in subsequent experiments.

### 2.2. Optimization of Power Output for External Irradiation

**Figure 2:**
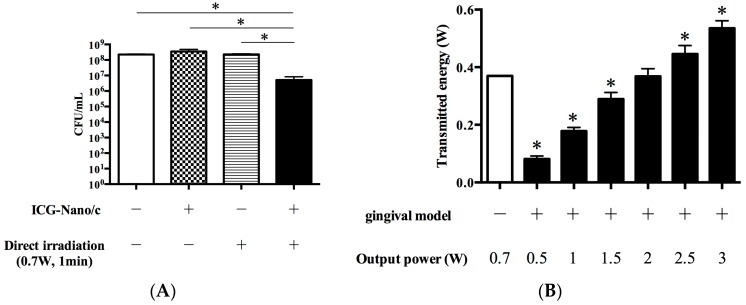
Optimization of power output for external irradiation. (**A**) Bactericidal effect of aPDT with ICG-Nano/c by direct irradiation with a diode laser. The diode laser emitted by the apparatus used in this study had a peak power output of 0.7 W and showed an appreciable aPDT effect by direct irradiation in combination with ICG-Nano/c; (**B**) Determination of optimal power output for external irradiation. Permeable energies transmitted through the gingival model at different power outputs (**black column**) were compared with the transmitted energy of direct irradiation with a power output of 0.7 W (**white column**), shown in B to have the required bactericidal efficacy. Data are the mean ± standard deviation. * *p* < 0.05 (*n* = 3).

**Figure A1:**
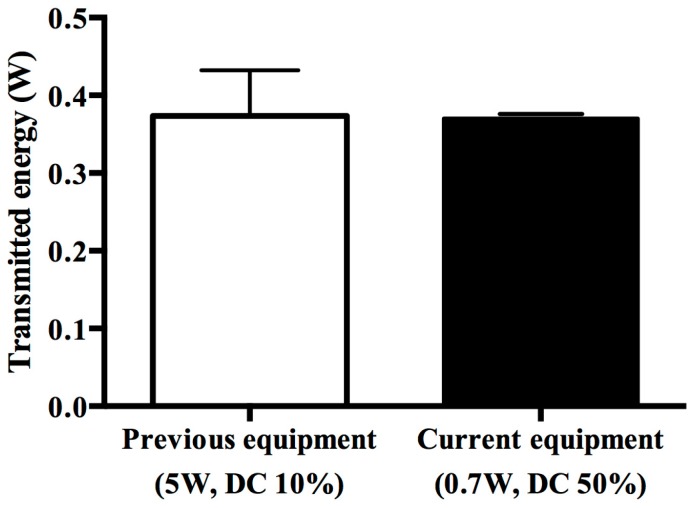
Revision of transmitted energies between laser equipment used in our previous study and the present study.

For trans-gingival aPDT to produce a sufficient inhibitory effect against bacteria, the permeable energy through the gingival model must be equal to or higher than that transmitted by direct irradiation. Direct irradiation by the diode laser apparatus used in the present study produced adequate bactericidal effects for aPDT at a peak power output of 0.7 W (Figure 2A, Figure A1). This 2 log_10_ reduction in bacterial viability was the same efficacy as that described in our previous report [17]. The transmitted energy of this direct irradiation condition detected by the power meter was ≈0.4 W (Figure 2B). An equivalent level of permeable energy was achieved by indirect irradiation through the gingival model at a peak power output of 2 W (Figure 2B).

### 2.3. Absorption of Permeable Energy by Photosensitizers

**Figure 3:**
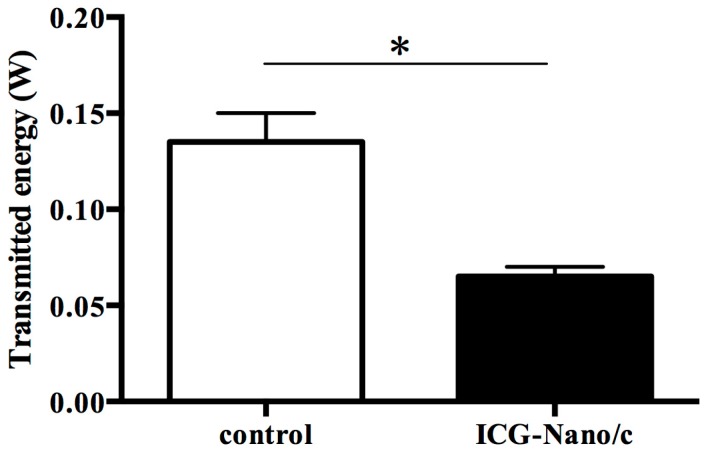
Absorption by the photosensitizer of permeable energy transmitted through the gingival model. The chart shows the energy penetration in different experimental scenarios. Control: gingival model and control medium; ICG-Nano/c: gingival model and ICG-Nano/c. Data are the mean ± standard deviation. * *p* < 0.05 (*n* = 3).

To ascertain if the light transmitted through the gingival model retained the ability to excite the photosensitizer, the absorption of permeable energy by ICG-Nano/c was investigated. Figure 3 shows that—After passing through the gingival model—Significantly less energy penetrates through the ICG-Nano/c solution layer than through the control medium. This result suggested that the energy penetrating through the gingival model was absorbed by the ICG-Nano/c liquid and, thus, could be used to excite the photosensitizer.

### 2.4. Comparison of Cooling Effects

**Figure 4:**
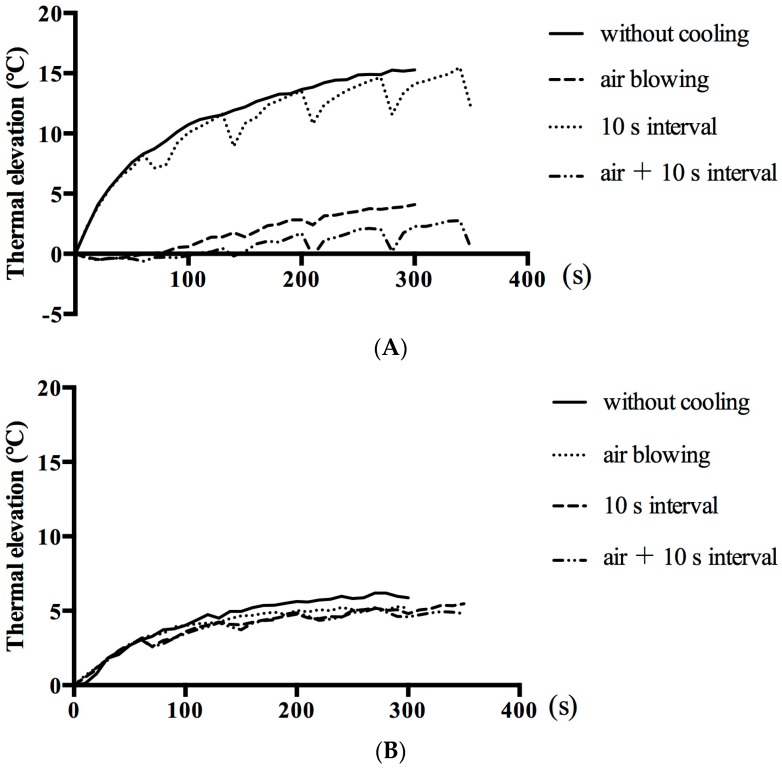
Temperature changes during external irradiation with different cooling paradigms. (**A**) Temperature changes at the surface of the gingival model; (**B**) Temperature changes in the ICG-Nano/c solution beneath the gingival model. The mean value of the highest temperatures within a heat distribution captured by thermography is indicated in polygonal lines (*n* = 3). Cooling strategies were air cooling (air blowing at 2 L/min) and/or intermittent irradiation (10 s break in radiation every 60 s).

Avoidance of hyperthermic damage to gingival tissue is of paramount importance to external irradiation methods because these require higher irradiation energy than conventional intra-pocket irradiation. Figure 4 shows a comparison of four cooling methods for the trans-gingival irradiation model. The highest rise in temperature on the surface of the gingival model was observed during continuous irradiation (15.27 °C increase after 5 min of irradiation), whereas intermittent irradiation with air cooling limited the increase to just 2.72 °C at 5 min (Figure 4A). Temperature changes in the ICG-Nano/c solution were also compared, with the smallest increase (4.94 °C at 5 min) recorded during intermittent irradiation with air cooling (Figure 4B).

### 2.5. Bactericidal Effect of aPDT with Laser Transmitted through the Gingival Model

**Figure 5:**
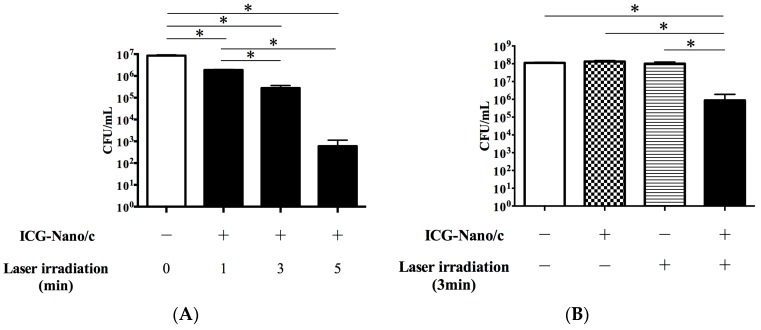
Bactericidal effect of aPDT with ICG-Nano/c in the external irradiation model. (**A**) Bactericidal effects with irradiation of different durations (1, 3, and 5 min) were shown by quantification of viable cell counts; (**B**) Bactericidal effects with or without ICG-Nano/c and laser irradiation for 3 min were shown by quantification of viable cell counts. *P. gingivalis* (10^8^ CFU/mL) mixed with ICG-Nano/c were irradiated intermittently (10 s pause every 60 s) by the diode laser at a peak power output of 2 W in a 50% duty cycle with air cooling. Data are the mean ± standard deviation. * *p* < 0.05 (*n* = 3).

The ultimate aim of the present study was to ascertain if aPDT with external irradiation showed a sufficient bactericidal effect against periodontal pathogenic bacteria to be considered for trans-gingival periodontal therapy in vivo. Using the conditions optimized above (2 W; 50% duty cycle with a pulse of 100 ms; intermittent irradiation with air cooling), we tested the bacterial viability after irradiation. Figure 5 shows post-irradiation colony-forming unit (CFU) counts on a logarithmic scale. The viability of *Porphyromonas gingivalis* was decreased significantly in all of the aPDT groups (1, 3, and 5 min) compared with the control group, and the efficacy of aPDT increased proportional to the duration of irradiation (Figure 5A). The most significant reduction in bacterial viability observed was on the order of 4 log_10_ (99.99% reduction) when the mixture was irradiated for 5 min. After irradiation for 3 min, bacterial viability was reduced by an order of 2 log_10_ (96.71%), equivalent to the aPDT effect of direct irradiation for 1 min reported previously [17]. External irradiation for 1 min produced a reduction of 78.12%. To confirm that this bactericidal effect was caused by aPDT, a culture of *P. gingivalis* mixed with or without ICG-Nano/c was irradiated by a diode laser for 3 min through the gingival model (Figure 5B). Bacterial viability was reduced significantly in the aPDT group, but not in the control, ICG-Nano/c-only, or laser-only groups.

## 3. Discussion

This report describes, for the first time, the bactericidal effectiveness of a new trans-gingival method of photoactivation that permits aPDT by laser irradiation from outside the periodontal pocket. Studies of laser irradiation of the gingival surface have focused on acceleration of healing in wounds or lesions. Here, we developed an irradiation method to transmit energy, in a trans-gingival manner, to activate a photosensitizer for aPDT. The advantages of this irradiation method for aPDT over conventional intra-pocket irradiation are: an operative procedure is facilitated; reduced invasiveness (and thus, less discomfort) due to no requirement for insertion of a laser probe into the periodontal pocket; and effective delivery of appropriate light energy into areas that would otherwise be inaccessible.

Blood-borne pigments such as hemoglobin, oxyhemoglobin, and bilirubin are major causes of absorbance of visible radiation in the dermis [22]. However, maintenance of a physiologic level of blood-borne chromophores in a gingival sample ex vivo is not possible because its blood supply is terminated upon removal from the living body. Myoglobin is an iron-containing porphyrin pigment, as are hemoglobin and oxyhemoglobin, and its absorption spectrum resembles that of hemoglobin. Therefore, we used muscle tissues (which contain myoglobin) as gingival models to mimic the wall of the periodontal pocket. Beef proved to be more effective than pork or chicken for the reduction of the permeable energy. Hence, laser light penetrating through these beef slices should be able to pass through actual gingiva. At a laser peak power output of 2 W, the power transmitted though the gingival model was ≈0.4 W. Anders et al. reported that the percentage transmission of light of 810 nm wavelength through the gastrocnemius muscle of rats was 7.42%, and through the overlying skin was 24.63% [25], data that are consistent with our results.

We calculated that ≥2 W of power output was required to transmit sufficient energy for trans-gingival aPDT. This output power is higher than that used in conventional aPDT (200–500 mW) [12,26], and increased the risk of causing histologic damage by hyperthermia. However, we suppressed this temperature increase in tissue to <3 °C using a protocol of intermittent irradiation with air cooling. Reports have suggested that irreversible reactions such as hyperthermal denaturation of tissue proteins occur at temperature increases of >20 °C, and that transient reversible changes are caused by temperature increases of >5 °C [27]. We conclude that the influence of temperature by this irradiation method is minimal and non-injurious, but that periodic application of a laser and continuous air cooling during irradiation are fundamental to hyperthermia suppression (because the temperature rise is >15 °C if they are not employed).

We confirmed that laser light penetrating through the gingival model retains its ability to excite the photosensitizer. Our data are in accordance with studies showing that a laser transmitted through tissue maintains its coherence [28]. To confirm this finding, we measured the absorbance of permeable energy by the ICG-Nano/c solution instead of detecting ROS generation directly because measuring ROS is technically very difficult due to its short lifetime (1 × 10^−6^ s).

Bacterial viability was reduced by an order of 2 log_10_ and 4 log_10_ after external irradiation for 3 and 5 min, respectively. The bactericidal effect of this irradiation method was equivalent to that described in our report on aPDT with ICG-Nano/c [17] and in other studies of aPDT reporting ICG-mediated inhibition of the planktonic *Staphylococcus aureus* and *Pseudomonas aeruginosa* cells with a maximum reduction of 99% [15]. Conversely, aPDT using other photosensitizing dyes produced more substantial effects. For instance, methylene blue excited with a 670 nm laser reduced the viability of *P. gingivalis* by an order of 7 log_10_ [29]. However, the ability of 670 nm-light to penetrate through the mucosa is lower than that of light at 810 nm and, thus, is not suitable for trans-gingival irradiation.

ICG has been used as a photosensitizer for photothermal therapy because it has higher calorific properties compared with other photosensitizers following laser irradiation [30,31], and the associated temperature increase may enhance its bactericidal effect. Kranz et al. reported that NIR irradiation of high concentration ICG heated a bacterial solution to >60 °C and caused bacterial inactivation due to this hyperthermic effect. They suggested that the generation of heat was thereby influenced by the concentration of the applied ICG and the total exposure value [26]. In our study, the temperature in the ICG-Nano/c solution was <5 °C above room temperature. This finding suggested that the thermal effect of ICG might not be responsible for its influence on bacterial viability and that its bactericidal effect might be caused predominantly by its stimulation of ROS. However, temperature measurement by thermography is associated with some limitations. A rapid change in temperature within 16 ms and changes in areas smaller than 270 × 270 μm could not be detected by the thermography system used in the present study. If a rapid momentary increase in temperature occurred immediately adjacent to the ICG/Nano-c solution, then exact prehension of this phenomenon would be very difficult. Therefore, the thermal effect for the bactericide could not be excluded.

In the present study, we described a new irradiation method for aPDT. This method was developed to produce bactericidal effects and levels of patient safety comparable with those of conventional methods of irradiation. Combination of a diode laser and ICG enabled the approach of sub-gingival areas through overlying tissue. The distinctive benefits of this irradiation method may be to gain access to deep tissue layers without damaging the tissue surface, and to improve therapeutic penetration into previously inaccessible periodontal areas. Future study is likely to further validate the application of other PDT protocols to treatment of profound lesions.

## 4. Materials and Methods

### 4.1. Bacterial Strain

*P. gingivalis* strain ATCC33277 was maintained by weekly subculture on blood agar plates in an anaerobic chamber at 37 °C. Then, *P. gingivalis* was inoculated into tryptic soy broth (Becton, Dickinson & Co., Cockeysville, MD, USA) supplemented with yeast extract (1 mg/mL), hemin (5 μg/mL), and menadione (1 μg/mL), and was cultured anaerobically to the mid-log phase at 37 °C. Cell numbers were measured in a spectrophotometer at 600 nm and diluted using culture medium to 0.1 optical density units (≈1 × 10^8^ CFU/mL) before use in the bactericidal assay.

### 4.2. Preparation of Photosensitizer-Loaded Nanospheres

An original photosensitizer, ICG-Nano/c was prepared by the emulsion solvent diffusion method in oil according to methods reported previously [17,32,33]. Briefly, ICG (Ophthagreen; Santen Pharmaceutical, Osaka, Japan), Poly(lactic-co-glycolic acid), and span80 solution dissolved in acetone and methanol was poured into an outer phase (2% Hexaglycerin-condensed ricinoleate containing triglyceride and *n*-hexane) at 2 mL/min with stirring at 35 °C under a vacuum. After removal of *n*-hexane and oil by centrifugation, the nanospheres were dispersed in a solution of 2% polyvinylalcohol/0.5% chitosan to coat the nanospheres with chitosan, and were then centrifuged at 26,690× *g* for 10 min at 4 °C. The sediment of nanospheres was suspended in mannitol solution. Finally, the suspension was frozen at −45 °C and lyophilized using a freeze-dryer for 48 h. ICG-Nano/c contained 5 mg/g ICG and the mean sphere size was 560 nm. Fresh solutions of ICG-Nano/c were prepared in the culture medium at 20 mg/mL and were used at a final concentration of 10 mg/mL.

### 4.3. Laser Application

The irradiation source used was a diode laser (LIGHTSURGE SQUARE; Osada, Tokyo, Japan) with a power output capacity of 3 W and a central wavelength of 810 ± 20 nm. Light was distributed by a fiber-optic applicator and the diameter of the fiber core was 600 µm. The spread angle of emitted light was 26°, which was defined by the incidence angle. The setting of the laser equipment used in this study was a repeated pulse mode with pulse width of 100 ms, 50% duty cycle, and variable peak power output (0.5–3 W, 4–24 W/cm^2^). The light probe was set 10 mm above the surface of the gingival model or suspension medium of the bacteria. The irradiation spot area was a circle of diameter 2.8 mm.

### 4.4. Requirements for Gingival Model

A gingival model was designed to fulfill the following requirements: (i) non-synthetic biological material; (ii) thicker than the thickest part of human gingiva and hard palate [34]; and (iii) containing myoglobin (as a proxy for blood-borne pigments such as hemoglobin, which accounts for most of the light absorbance in the dermis [22]). The selected candidate models were 3-mm slices of fresh beef, pork, and chicken, and were set up on a power meter (LASERSTAR; Ophir Optronics Solutions, Jerusalem, Israel). The permeable energies through the gingival models were compared at different peak power outputs.

### 4.5. Determination of the Optimal Power Output for External Irradiation

To investigate the power output necessary to deliver sufficient energy in a trans-gingival manner, the permeable energy through the gingival model was measured by a power meter and was compared with the transmitted energy from direct irradiation required for effective aPDT with ICG-Nano/c. Various peak power outputs were used for this experiment, all with an irradiation duration of 1 min.

### 4.6. Confirmation of Light Absorption by Photosensitizers

ICG-Nano/c liquid (200 μL; final concentration, 10 mg/mL) was placed into each well of a 96-well plate set over a power meter; a gingival model tissue slice was mounted on top of the plate. Culture medium was prepared in the same manner as that described for controls. The diode laser was irradiated from above the gingival model for 3 min and the energy absorption by the ICG-Nano/c solution was measured.

### 4.7. Comparison of Cooling Methods

The four cooling strategies were as follows: (i) no cooling (continuous irradiation); (ii) intermittent irradiation; (iii) continuous irradiation with air cooling; (iv) intermittent irradiation with air cooling. In intermittent irradiation, the laser irradiation from above the gingival model was suspended for 10 s every 60 s. Air cooling was done by blowing air onto the surface of the gingival model at 2 L/min using an air pump (silent-β60; As One, Osaka, Japan). Temperature distribution in the gingival model and ICG-Nano/c liquid in a 0.2-mL tube were captured by thermography (initial temperature, 26 °C; Thermo GEAR G100; Nippon Avionics, Tokyo, Japan). The shooting distance of thermography was 15 cm from the surface of the gingival model or tube containing the ICG-Nano/c liquid. The minimum detection limit on this shooting distance was 270 μm. The frame time was 60 Hz and heat images were recorded every 3 s.

### 4.8. Bactericidal Assay

Bacterial suspension (100 μL of a 1 × 10^8^ CFU/mL suspension in liquid culture medium) was mixed with an equal volume of ICG-Nano/c liquid in a 0.2 mL tube. Then, this mixture was mounted under the gingival model and irradiated by a diode laser from 10 mm above the surface of the gingival model. After these procedures, the mixed suspension was diluted from 10^−4^ to 10^−7^ and plated onto blood agar plates. CFU were counted after 7 days of anaerobic culture.

### 4.9. Statistical Analysis

Data were analyzed by normality tests (Shapiro–Wilk and Kolmogorov–Smirnov): a normal distribution was confirmed. Thus, in comparison of the irradiation conditions and CFU counts, data were analyzed with a parametric test (Dunnett’s or Tukey test) by SPSS v15.0 (IBM, Armonk, NY, USA), with significance accepted at *p* < 0.05.

## 5. Conclusions

We presented a new strategy for an irradiation method with photosensitizing nanospheres, ICG/Nano-c, by trans-gingival delivery of laser energy in vitro. Results showed that the viability of a periodontal pathogen was reduced significantly by laser irradiation on ICG/Nano-c through a gingival model. The temperature increases of the gingival model and ICG/Nano-c liquid were suppressed sufficiently with air cooling, to avoid thermal damage to periodontal tissues. However, further studies are needed to reveal the mechanisms of bacterial inactivation (including the possibility of a thermal effect) and to ensure the safety of this method for clinical application.

## Appendix A

The laser equipment and irradiation setting used in the present study were different from those in our previous study (which showed a sufficient bactericidal effect). Hence, transmitted energies were revised by measurement of transmitted energies using a power meter. The white column denotes the transmitted energy of the equipment and settings of our previous study (repeated pulse with a pulse width of 100 ms, peak power output of 5 W, 10% duty cycle). The same level of transmitted energy was observed with the setting of a pulse width of 100 ms, peak power output of 0.7 W, and 50% duty cycle for the equipment used in the present study (black column). Data are the mean ± standard deviation (*n* = 3). DC, duty cycle.
